# How successful was the use of a community of practice for the implementation of evidence-based practices for heart failure within the United States Department of Veterans Affairs: Insights from a formative evaluation

**DOI:** 10.1186/s12961-022-00880-9

**Published:** 2022-07-08

**Authors:** Anju Sahay, Brian S. Mittman, Parisa Gholami, Shoutzu Lin, Paul A. Heidenreich

**Affiliations:** 1 United States Department of Veterans Affairs, Veterans Affairs Palo Alto Health Care System, 3801 Miranda Avenue, Palo Alto, CA 94304 USA; 2 Department of Research & Evaluation, Kaiser Permanente Southern California, 100 South Los Robles Avenue, 3rd Floor, Pasadena, CA 91101 USA; 3 Department of Medicine, Stanford University School of Medicine, Stanford, CA 94305 USA

**Keywords:** Community of Practice, Social network, Evaluation, Health policy, Health systems, Formative evaluation, Quality improvement

## Abstract

**Background:**

Communities of Practice (CoPs) are a promising approach to facilitate the implementation of evidence-based practices (EBPs) to improve care for chronic conditions like heart failure (HF). CoPs involve a complex process of acquiring and converting both explicit and tacit knowledge into clinical activities. This study describes the conceptualization, creation, capacity-building and dissemination of a CoP sustained over 9 years, and evaluates its value and impact on EBP.

**Methods:**

In July 2006, a CoP called the Heart Failure Provider Network (HF Network) was established within the United States Department of Veterans Affairs (VA) with the overarching goal of improving the quality of care for HF patients. We assessed (formative) the HF Network in terms of its various activities (inputs) and proximal impacts (mediators) at the individual level, and its distal impacts (outcomes) at the site level including implementation of new/improved EBPs at the systemwide level.

**Results:**

The HF Network membership grew steadily over the 9 years. The CoP has involved a total of 1341 multidisciplinary and multilevel members at all 144 VA Health Care Systems (sites). Most members were practising clinicians (*n* = 891, 66.4%), followed by administrators (*n* = 342, 25.5%), researchers (*n* = 70, 5.2%) and others (*n* = 38, 2.8%). Participation was assessed to be “active” for 70.6% versus “passive” for 29.4% of members. The distribution of active members (clinicians 64.7%, administrators 21.6%) was similar to the distribution of overall membership.

**Conclusions:**

Survey respondents perceived the HF Network as useful in terms of its varied activities and resources relevant for patient care. Strong evidence shows that these members, particularly those who considered themselves influential in improving quality of care, noted multiple benefits of membership, which included confirmation of their own clinical practices, evidence-based changes to their practice and help in understanding facilitators and barriers in setting up or running HF clinics and other programmes. Such CoPs have strong impacts on the quality of care being delivered for both mandated and non-mandated initiatives.

## Background

Proactive management of knowledge is today seen as a key strategy to ensure the performance and success of organizations or systems [1]. Those running well-organized health research systems are likely to be alert for ways in which they might increase the quality of the services they provide and address any problems identified. This is important because the efficiency of the research system can have a major impact on how long it takes for new treatments to be developed and reach patients. Continuous improvement is a cycle, an activity that is done constantly and over time, rather than an act or linear activity [2]. In a 2020 systematic review conducted by Hill et al. [3], where continuous quality improvement (QI) was found to be effective, collaboration and communication between healthcare professionals appeared important. A major challenge to integrating evidence into practice for conditions such as heart failure (HF) is that it involves a complex process of acquiring and converting both explicit and tacit knowledge into clinical activities. Explicit knowledge is codified information such as peer-reviewed articles, rules and guidelines which can be readily shared through written documents and other communication channels [4]. Tacit knowledge, in contrast, requires intensive social interaction and exchange. Although both forms of knowledge are critical for effective professional practice and healthcare delivery, most policy, practice and research activity to improve quality of care emphasizes explicit knowledge. Recent interest and expanded research activity examining Communities of Practice (CoPs) and related concepts, however, are beginning to redress this imbalance. Auer and colleagues postulate that CoPs enable the diverse wealth of knowledge embedded in people, local conditions and special circumstances to flow from practice domain groups to programme and service areas, and into the larger system where it can effect organizational change [5].

### Communities of Practice (CoPs)

CoPs have been used in the health sector to support professional practice change [6]. They enable the diverse wealth of knowledge embedded in people, local conditions and special circumstances to flow from practice domain groups to programme and service areas, and into the larger system where it can effect organizational change [5]. In 1991, Lave and Wenger [7] developed the concept of the CoP. They suggested that learning takes place through social relationships rather than through the simple acquisition of knowledge. These informal communications became the means for sharing information for improving practice and generating new knowledge and skills. In 1998, Wenger [8] proposed three CoP dimensions: mutual engagement (the interaction between individuals that leads to the creation of shared meaning), joint enterprise (the process in which people are engaged and work together towards a common goal), and a shared repertoire (the common resources and jargon that members use to negotiate meaning within the group). Later, in 2002, Wenger et al. refined the description of CoPs as “groups of people who share a concern, a set of problems, or a passion about a topic, and who deepen their knowledge and expertise in this area by interacting on an ongoing basis” [9]. They identified three essential characteristics of CoPs: (1) the “'domain” creates common ground (i.e. the minimal competence that differentiates members from nonmembers) and outlines the boundaries that enable members to decide what is worth sharing and how to present their ideas; (2) the “community” creates the social structure that facilitates learning through interactions and relationships with others; and (3) the “practice” is the specific knowledge that the community shares, develops and maintains. Wenger and colleagues purported that a well-developed CoP group (i.e. when the three elements work well together) provides an environment that facilitates learning and knowledge development. They suggested that an ideal CoP group should include a leader(s)/champion(s), a facilitator(s), a core group of experts who regularly interact with the group, and a dedicated group of members with varying levels of expertise. Their work suggested that organizations can engineer and cultivate CoPs to enhance their competitiveness [9]. According to Bertone et al. [1], CoPs represent a potentially valuable tool for producing and sharing explicit knowledge, as well as tacit knowledge and implementation practices.

Li and colleagues have argued that the literature is less clear on how to foster the three CoP elements, especially at the early stage [12]. To improve their understanding about the use of the CoP concept, they conducted a research synthesis project to explore how the concept was operationalized in the business and health sectors. Findings showed that among shared characteristics of CoPs in business and healthcare, learning and sharing information through socialization appeared to be the central characteristic of the CoP groups. To varying degrees, all CoPs demonstrated four characteristics: social interaction among members (interaction of individuals in formal or informal settings, in person or through use of communication technologies); knowledge sharing (process of sharing information that is relevant to the individuals involved); knowledge creation (process of developing new ways to perform duties, complete a task, or solve a problem); and identity-building (process of acquiring a professional identity, or an identity of being an expert in the field).

CoPs have been described as a type of informal learning organization and are gaining popularity in the health sector. Some CoPs resemble an informal network, where the goal and structure of the group is loosely defined, and others are similar to support groups, where the main goal is to enhance self-efficacy.

### Health impact of CoPs

One version of a CoP, known as a clinical community, is an emerging approach to QI to which several large-scale projects have attributed some success [10]. Clinical communities evolved from previous approaches that used collaboration to achieve improvement, such as clinical networks and collaboratives to improve quality of care, patient safety and value across the health system. The collaborative spirit of the communities embraced interdisciplinary membership and engaged in team-building activities and facilitated discussions, met monthly, and were encouraged to meet in person to develop relationships and build trust. Healthcare organizations can promote knowledge creation and utilization by chronic patients through the introduction of a virtual, private, disease-specific patient community [11]. Such patient-centred healthcare organizations can employ the virtual community to direct and support the empowerment of chronic patients in their care. While there is evidence for improved process of care, there is limited evidence to show that CoPs affect healthcare outcomes. In their 2009 literature review from 1991 to 2005, Li and colleagues [12] found no studies to show improvements in health outcomes of CoPs in the health sector. In another comprehensive review of studies from 1990 to 2009, Ranmuthugala [13] noted that little is known about the organizational processes that lead to successful creation of knowledge-based structures such as CoPs.

### Medical education

In terms of its implications for medical education, Cruess and colleagues [14] reported that CoPs could serve as the foundational theory, and other theories could provide a theoretical basis for the multiple educational activities that take place within the community, thus helping create an integrated theoretical approach. CoPs can guide the development of interventions to make medical education more effective and can help both learners and educators better cope with the complexity of medical education.

### Heart Failure Provider Network

In July 2006, the United States Department of Veterans Affairs (VA)’s Chronic Heart Failure (CHF) Quality Enhancement Research Initiative (QUERI) established a CoP consisting of VA members to improve the quality of care provided to HF patients throughout the VA Health Care System. This multidisciplinary CoP is called the Heart Failure Provider Network (referred to as HF Network).

#### HF Network goals

The overarching goal for the HF Network is to facilitate knowledge exchange of evidence-based practices (EBPs) and strategies for improving quality of care for HF patients. The specific HF Network goals are as follows:

1. Share evidence-based HF programmes.
2. Understand and help resolve barriers to and facilitators of implementation.
3. Establish collaborations/networking.
4. Disseminate findings and implement QI projects.
5. Provide opportunities to identify/involve opinion leaders and/or local champions.

The HF Network includes members at all VA sites interested in improving HF care. It was rolled out at the national level and initiated with a single email to all known chiefs of medicine and chiefs of cardiology at the VA sites. They were asked to forward the invitation to all interested VA staff. Those expressing interest were sent an email invitation describing the HF Network, including its purpose, opportunities to present and next scheduled meeting. Membership grew based on peer/provider recommendations, VA newsletters and VA websites. Existing members may discontinue their membership at any time. Membership was voluntary, with members choosing which HF Network activities to attend. The leadership of the HF Network was based at the VA Palo Alto Health Care System and they organized all the HF Network activities.

The objective of this article is to describe a formative evaluation of the HF Network.

## Methods

### Conceptual frameworks for the evaluation of CoPs

McKellar et al. [15] reviewed evaluation frameworks for CoPs and found that strong claims about generalizability could not be made with limited applications of the frameworks. Richard et al. developed a conceptual model to evaluate an initiative based on a CoP strategy. This model was based on theories of work-group effectiveness and organizational learning and can be adapted by evaluators who are increasingly called upon to illuminate decision-making about CoPs [16]. The model took its strength from two improvements over the traditional input–process–output models. First, it used the term “mediation” to explain the transformation of its inputs into outcomes. Further, due to the feedback loops, it showed that outcomes would have an impact on organizational learning and practices that would necessarily affect individual and group characteristics.

### Conceptual framework for the evaluation of the HF Network as a CoP

**Fig. 1:**
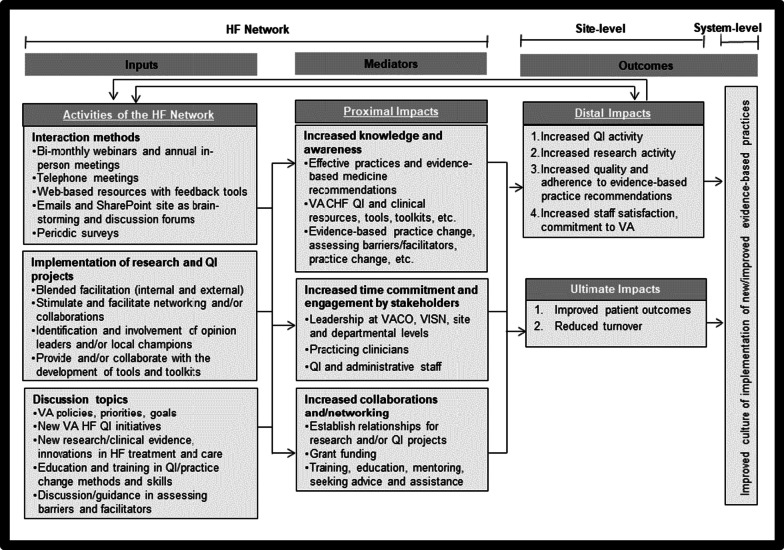
Conceptual framework for the evaluation of the HF Network as a CoP

Based on McKellar et al.’s approach [15], we have conceptualized the formative evaluation of the HF Network. Figure 1 highlights the HF Network’s conceptual framework for the evaluation in terms of its various activities (inputs), proximal impacts (mediators) at the individual level, and its distal and ultimate impacts (outcomes) on the implementation of new/improved EBPs at the sites and system-wide.

### Evaluation of the HF Network

Our evaluation of the HF Network can be considered a formative evaluation. The aim of the evaluation is to identify areas and approaches for making improvements in the activities of the HF Network. Stetler et al. [17] defined formative evaluation as “a rigorous assessment process designed to identify potential and actual influences on the progress and effectiveness of implementation effort. Formative evaluation enables researchers to explicitly study the complexity of implementation.” Both qualitative and quantitative methods were used.

### Assessment of the HF Network

#### Membership

Member roles were categorized as follows: leadership (VA Central Office (VACO)/Veterans Integrated Service Network (VISN) or regions, sites/departmental), practising clinicians (physicians, nurse practitioners, physician assistants, nurses, pharmacists and psychologists), QI and administrative staff (quality management and administrators), researchers and others.

#### “Active” participation

We have defined “active” versus “passive" participation. To qualify as an “active” participant, the member should have participated at least one of the four HF Network activities: (1) attended and/or presented during the bimonthly web-based meeting, (2) attended the annual in-person meeting, (3) sent request for slides, or (4) completed surveys. All remaining members were “passive” participants.

#### Activities of the HF Network

For our purpose, we chose to map Li’s four characteristics of CoP groups’ [4] work to the four categories of the HF Network activities: shared ways of engaging, resources, research/QI activities and relationships (Table 1).

**Table 1:** Mapping of Li’s characteristics of CoP groups to HF Network activities

HF Network activities	Li’s characteristics of CoP groups			
	Social interaction	Knowledge-sharing	Knowledge creation	Identity-building
*Shared ways of engaging*				
Bimonthly web-based meetings with conference calls	**X**	**X**	**X**	**X**
Annual in-person meeting	**X**	**X**	**X**	**X**
Surveys—email text and web-based links				
Email exchange	**X**	**X**	**X**	
Non-mandated forum of VA SharePoint site to exchange ideas	**X**	**X**	**X**	
Revise CHF QUERI Strategic Plan		**X**		
Networking	**X**			**X**
*Resources*				
HF Programmes		**X**	**X**	
HF Provider Toolkit		**X**	**X**	
HF tools		**X**	**X**	
Patient and caregiver education materials		**X**	**X**	
Funds for projects		**X**	**X**	
*Research/QI activities*				
Expand research activities	**X**	**X**	**X**	**X**
Expand QI initiatives	**X**	**X**	**X**	**X**
Recruit sites to conduct research and/or QI initiatives	**X**	**X**	**X**	**X**
Formative evaluation of HF Network	**X**	**X**		**X**
*Relationships*				
Collaborations	**X**	**X**	**X**	**X**
HF experts	**X**	**X**	**X**	**X**
New local opinion leaders and champions	**X**	**X**		**X**
Development of subspecialty networks	**X**	**X**	**X**	**X**

We have used the HF Network to disseminate results of randomized trials (e.g. clinical reminders for beta-blocker use) and to facilitate the implementation of national QI initiatives such as the VA Hospital-To-Home (VA H2H) initiative to reduce readmissions for Veterans with HF [18]. In collaboration with the members, we also developed an online HF Provider Toolkit for better management by members [19].

We tracked four specific activities to determine members’ “active” participation in the HF Network. The first, and most widely attended, was the bimonthly web-based meetings with conference calls. During the web-based meetings the moderators share announcements and updates, which were typically followed by two presentations made by members of the HF Network and guests (both VA and non-VA). The second was an annual in-person meeting. The third tracked activity was periodic online surveys to HF Network members. Those surveys queried sites on the presence of local QI projects and members’ views on VA goals related to the care of Veterans with HF. The fourth tracked activity was soliciting members of the HF Network to apply for funding for implementation projects from the VA’s QUERI programme funding as well as CHF QUERI core funds.

#### Survey

Six years after initiation, we conducted an evaluation to assess the strengths and weaknesses of the HF Network using a cross-sectional web-based survey of members (*n* = 878). This survey asked respondents questions about their participation in the activities, how helpful the various activities of the HF Network were, how beneficial participation in the network was, whether participation had an influence on improving the care of patients and, if applicable, reasons for not participating. The survey response rate was 24.9%.

#### Qualitative analysis: phone interviews

We used stratified purposeful sampling (*n* = 18) to identify key participants to conduct semi-structured phone interviews. All participants were members of the HF Network and were practising clinicians (physicians *n* = 10 and nurses *n* = 7) or VACO/VISN leadership (*n* = 1). Each participant belonged to a separate site and participated at varying levels in the HF Network (None/Low = 4, Moderate = 9 and High = 5). All interviews were audio-recorded and then transcribed.

### Site level

#### Setting/sites

We identified a total of 124 participating VA sites with HF Network members over the 9 years. We grouped sites by member participation (over years 1–4) into three levels: “none/low” (members at these sites participated in no or single activity; *n* = 47), “moderate” (members at these sites participated in 2–3 activities; *n* = 36) and “high” (members at these sites participated in 4 or more activities; *n* = 41).

#### Outcome measures

The quality indicator outcomes were 30-day mortality after admission, death at 1 year after readmission and all-cause admission after 30 days. We also examined guideline-recommended process of care measures in those with depressed left ventricular ejection fraction (LVEF) < 40%: use of beta blockers, use of angiotensin-converting enzyme (ACE) inhibitors and use of aldosterone antagonist. These data were obtained through linkages with quality data from chart reviews (medications and LVEF) and administrative data (mortality and hospitalization).

### Statistical analysis

#### Member level: survey

The HF Network database was created using Microsoft Access, and we tracked member role, membership period, years of membership and participation in activities. Categorical responses were compared using chi-square tests. Survey data were analysed using IBM SPSS Statistics version 21 software [20].

#### Member level: phone interviews

Interview data were analysed by the qualitative research team using de-identified verbatim interview transcripts entered into Atlas.ti [21] (a qualitative data management system) and coded by a trained analyst. Interviews were analysed by two qualitative researchers familiar with the topic using an emergent, thematic approach based on the tenets of grounded theory [22, 23]. A codebook was developed iteratively using feedback from members of the coding and research team until consensus on the codebook was reached. Core categories were identified, defined and operationalized to examine congruent, divergent and conflicting themes. Inter-coder reliability was considered as achieving a kappa statistic of 0.65 and above, or what Landis and Koch [22] describe as a “substantial” level of agreement.

#### Site-level analysis

All analyses for site-level data were conducted using Stata 11.0 software [24]. A *P* value of < 0.05 was considered statistically significant.

## Results

Following the format described in the Methods section, below we have first focused on the formation of the HF Network at the member-level. Based on the study database, we have provided descriptions of all members, sustainability in membership over the 9 years, member roles and their participation in the HF Network. Next, based on the quantitative data obtained through surveys and qualitative data obtained through phone interviews, we have provided results focused on the assessment of the HF Network both at the individual (member) level and site level.

### Formation of the HF Network

#### Member level

##### Description of all members (July 2006–June 2015)

As seen in Table 2, over the 9 years the HF Network had a total of 1341 members from 143 VA sites. Among them, as of June 2015, there were 930 current members, 145 past members who opted out for reasons such as change in work role or work overload, and 266 members who left the VA (Table 2).

**Table 2:** Characteristics of all HF Network members

Member role	Membership status (July 2006–June 2015)			
	Current member *N* (%)	Past member: (opted out) *N* (%)	Past member: (left VA) *N* (%)	Total *N* (%)
*Administration*				
Site/departmental leaders	159 (11.9)	27 (2.0)	24 (1.8)	210 (15.7)
VACO/VISN leaders	43 (3.2)	11 (0.8)	12 (0.9)	66 (4.9)
Other administrators	28 (2.1)	3 (0.2)	5 (0.4)	36 (2.7)
Quality management staff	23 (1.7)	3 (0.2)	4 (0.3)	30 (2.2)
*Practising clinicians*				
Physicians	255 (19.0)	43 (3.2)	110 (8.2)	408 (30.4)
Nurses	178 (13.3)	33 (2.5)	56 (4.2)	267 (19.9)
Nurse practitioners	95 (7.1)	6 (0.4)	20 (1.5)	121 (9.0)
Pharmacists	52 (3.9)	5 (0.4)	13 (1.0)	70 (5.2)
Physician assistants	11 (0.8)	3 (0.2)	3 (0.2)	17 (1.3)
Psychologists	6 (0.4)	1 (0.1)	1 (0.1)	8 (0.6)
*Researchers*	57 (4.3)	3 (0.2)	10 (0.7)	70 (5.2)
*Others*	23 (1.7)	7 (0.5)	8 (0.6)	38 (2.8)
TOTAL	930 (69.4)	145 (10.8)	266 (19.8)	1341 (100.0)

#### Sustainability of membership over 9 years

**Fig. 2:**
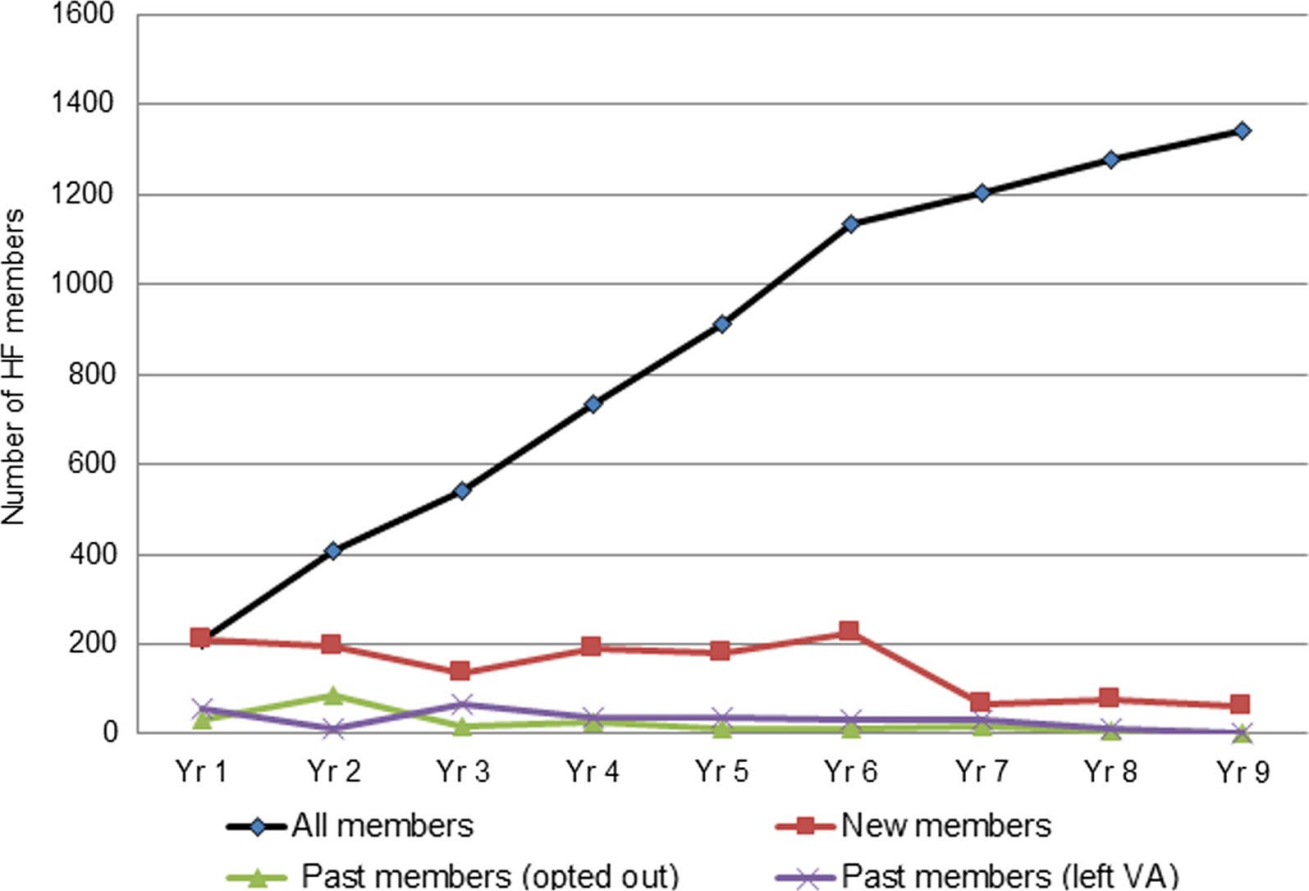
Sustainability of HF Network membership per year shown for all, new, past (opted out) and past (left VA) members

As seen in Fig. 2, membership in the HF Network was highly sustained. Membership increased steadily particularly in year 1 (*n* = 210), year 5 (*n* = 911) and year 9 (*n* = 1341). The highest number of new members joined the HF Network in year 6 (*n* = 224), followed by year 1 (*n* = 210). Over the years, some members also left the HF Network, with the highest number leaving in year 2 (*n* = 90) followed by year 1 (*n* = 34), while some members left the VA, with the highest number in year 3 (*n* = 68) and lowest number in year 9 (*n* = 3).

#### Member roles

**Fig. 3:**
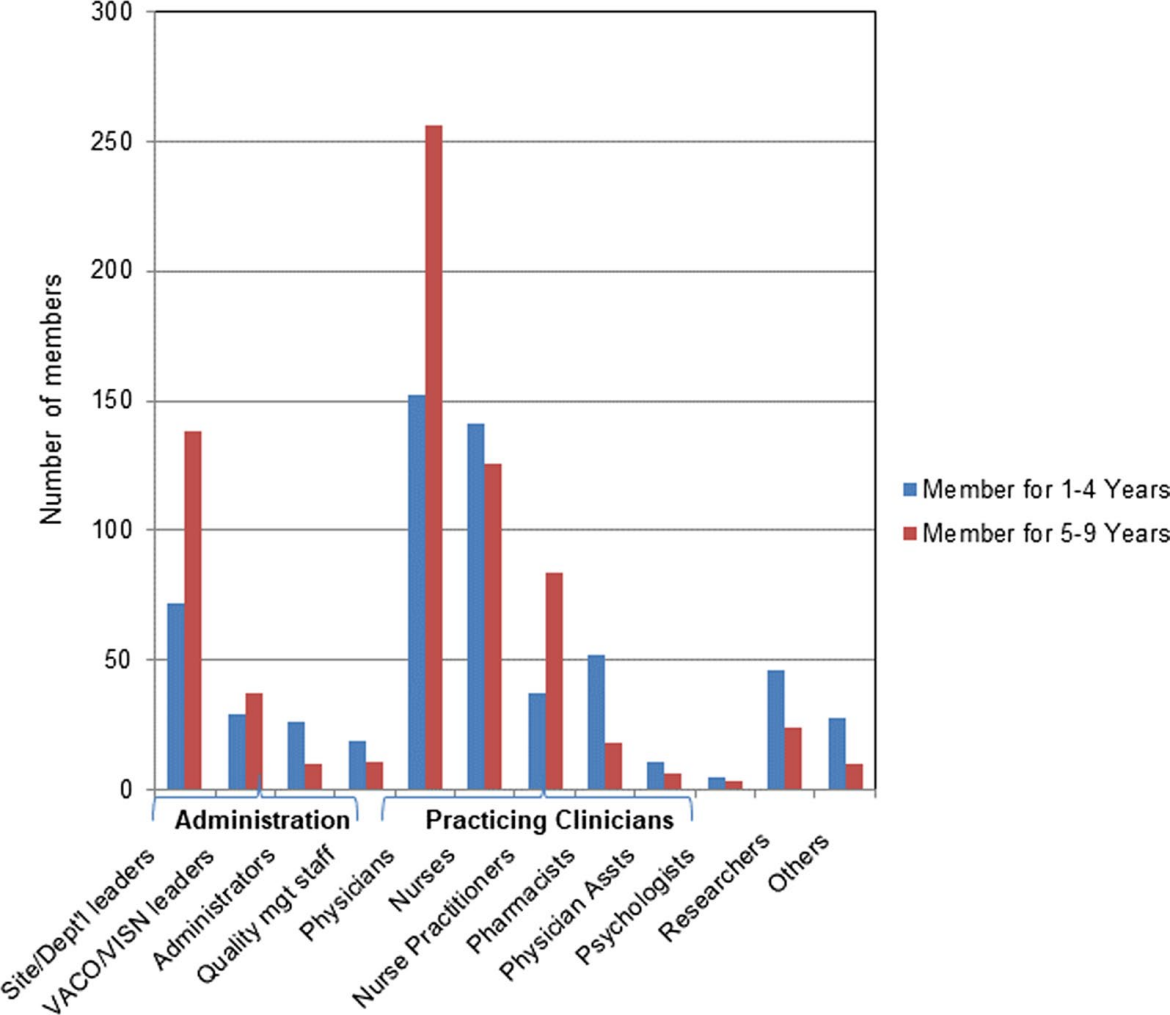
Member role and years of membership in HF Network

Figure 3 shows the distribution of member roles as highest for practising clinicians (*n* = 891, 66.4%), followed by administration (*n* = 342, 25.5%), researchers (*n* = 70, 5.2%) and others (*n* = 38, 2.8%). Among the practising clinicians, the highest membership in the HF Network was for physicians (*n* = 408, 30.4%), followed by nurses (*n* = 267, 19.9%).

#### “Active” participation

**Fig. 4:**
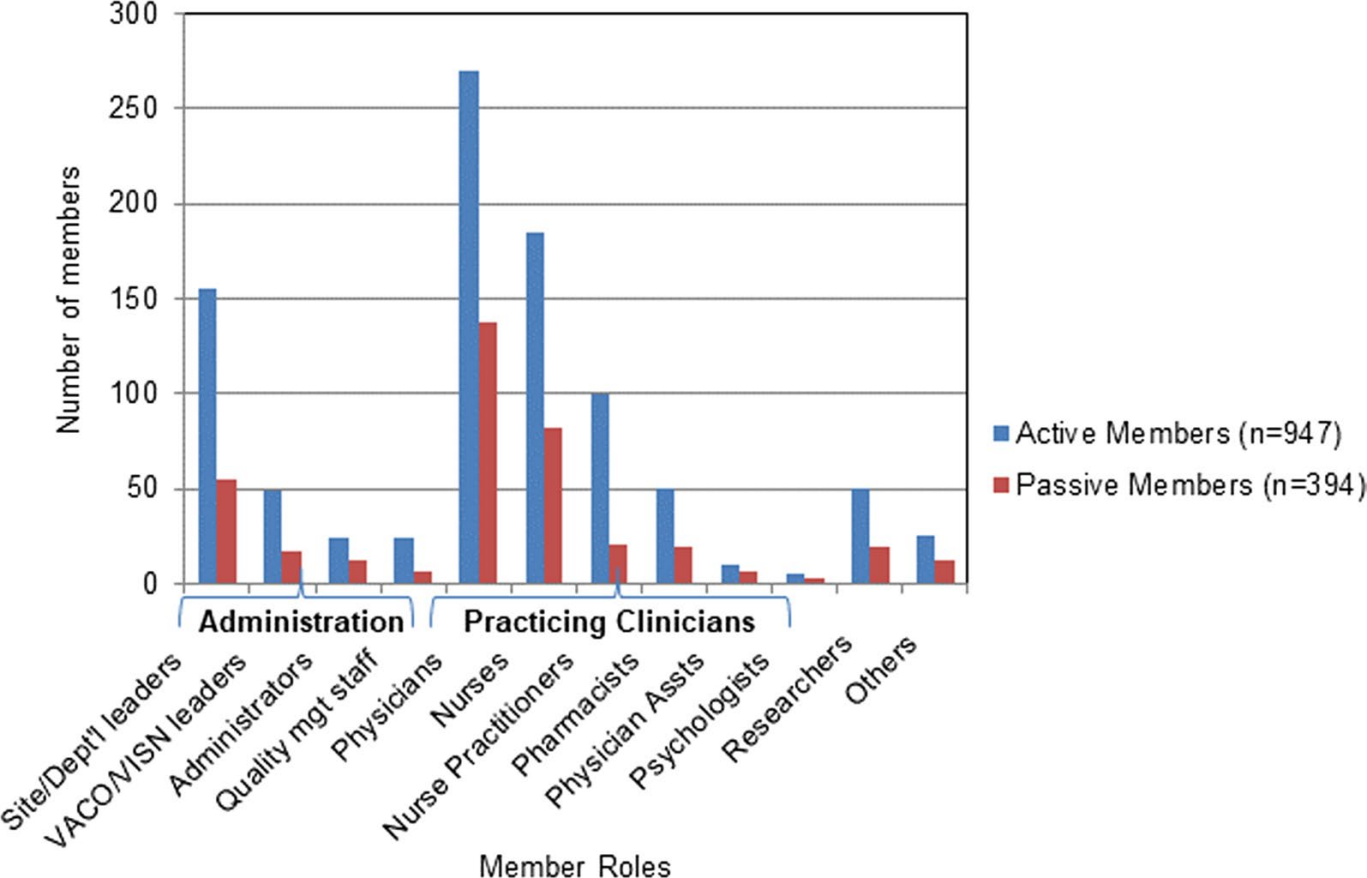
Member role and active versus passive members of the HF Network

Figure 4 shows that among the members, there were “active” participants (*n* = 947, 70.6%, range 1–23 activities) and “passive” participants (*n* = 394, 29.4%, range 0–0 activities).

**Fig. 5:**
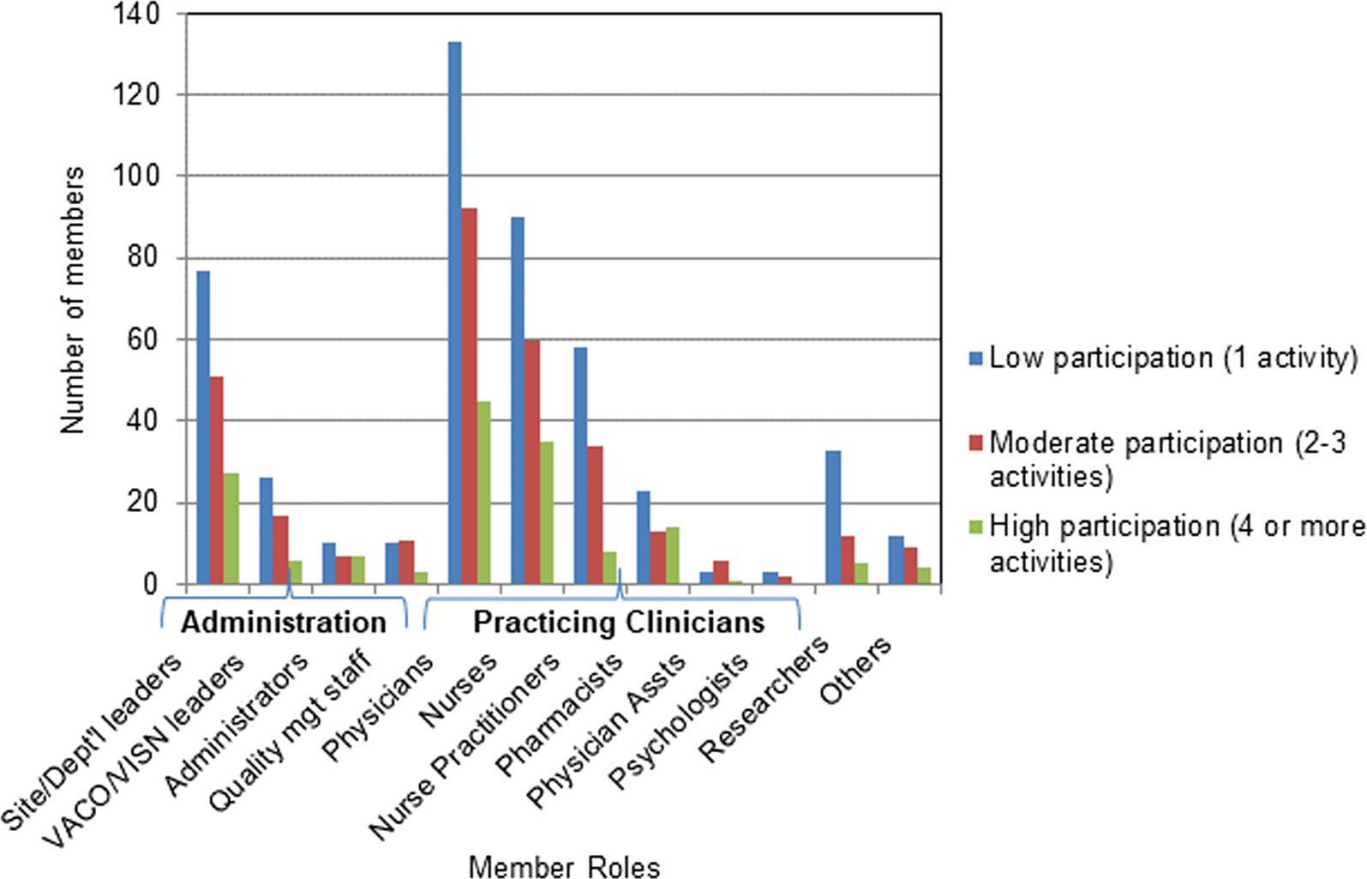
Member role and level of active participation in the HF Network

Focusing solely on active members, Fig. 5 shows that they participated in HF Network activities at low (one activity), moderate (2–3 activities) and high (four or more activities) levels. As seen, practising clinicians participated most frequently in HF Network activities (total *n* = 1518, 64.7%), and among them the highest participation was seen for physicians (total *n* = 668, 44.0%), followed by nurses (total *n* = 466, 30.6%). The second-highest level of participation was observed for administration (*n* = 507, 21.6%), and among them, compared to VACO/VISN leadership, higher participation was seen for the site/departmental leaders (n = 406, 80.0%). The remaining two categories participated to a much lesser extent.

### Assessment of the HF Network

#### Survey findings

The survey was emailed to 878 members who had been a member of the HF Network for at least 6 months. Table 3 provides descriptions of roles of all respondents. The roles of the respondents were consistently representative of the roles of the total HF Network members. Highest representation was observed for the practising clinicians (respondents *n* = 144, 65.6% versus all *n* = 891, 66.4%) followed by administration (respondents *n* = 54, 24.7% versus all *n* = 276, 20.6%), then researchers (*n* = 17, 7.8% versus *n* = 70, 5.2%) and others (n = 4, 1.8% versus *n* = 8, 0.6%). The survey response rate was 24.9% (*n* = 219).

**Table 3:** Characteristics of members: responded to survey and all members

Member role	Survey respondents (members) N (%)	All HF Network members N (%)
*Administration*		
VACO/VISN leaders	12 (5.5)	66 (4.9)
Site/departmental leaders	35 (16.0)	210 (15.7)
Quality management staff	6 (2.7)	30 (2.2)
Administrators	1 (0.5)	36 (2.7)
*Practising clinicians*		
Physicians	50 (22.8)	408 (30.4)
Nurse practitioners	43 (19.6)	121 (9.0)
Physician assistants	4 (1.8)	17 (1.3)
Nurses	32 (14.6)	267 (19.9)
Pharmacists	15 (6.8)	70 (5.2)
Psychologists	0 (0.0)	8 (0.6)
*Researchers*	17 (7.8)	70 (5.2)
*Others*	4 (1.8)	8 (0.6)
Total (%)	219 (100)	1341 (100)

Involvement with QI projects/programmes at own site		
*Type of QI role*		
Formal role	34 (23.1)	
Informal role	35 (23.8)	
Both formal and informal roles	78 (53.1)	
*Member QI role specific to*		
HF-related QI projects	74 (50.3)	
Non-HF-related QI projects	14 (9.5)	
All types of QI projects	58 (39.5)	
Total (%)	147 (67.7)	

Also, shown in Table 3 is that 67.7% of the respondents were involved with QI projects/programmes at their own site. Among them 53.1% respondents reported being involved in both formal and informal roles, and the remaining respondents were involved either in formal (23.1%) or informal (23.8%) roles in the projects. Further, 50.3% of the respondents were involved with HF-related projects while 39.5% of them were involved with all types of QI projects (HF-related and non-HF-related).

Respondents were also asked whether the five goals of the HF Network were of particular interest to them, and whether they perceived these goals as being achieved by the HF Network at least to a moderate extent.

Table 4 shows that a majority of the respondents expressed significant interest in four out the five goals (range 85.8–95.4%), and most of them reported that all goals had been achieved at least to a moderate extent (range 82.4–97.3%).

**Table 4:** HF Network goals as perceived by members

Goals	Goals of particular interest to members *N* (%)	Goals achieved by HF Network at least to a moderate extent *N* (%)
Share evidence-based HF programmes and updates in HF care	219 (95.4)	219 (97.3)
Understand the context in providing HF care (e.g. site, culture, leadership style, HF programme)	219 (85.8)	219 (90.6)
Learn about barriers and facilitators to improving HF care	219 (92.2)	219 (91.2)
Establish collaboration and/or network among members of the HF Network	219 (88.6)	219 (90.0)
Provide opportunities to identify/involve local champions at sites	219 (73.5)	219 (82.4)

**Fig. 6:**
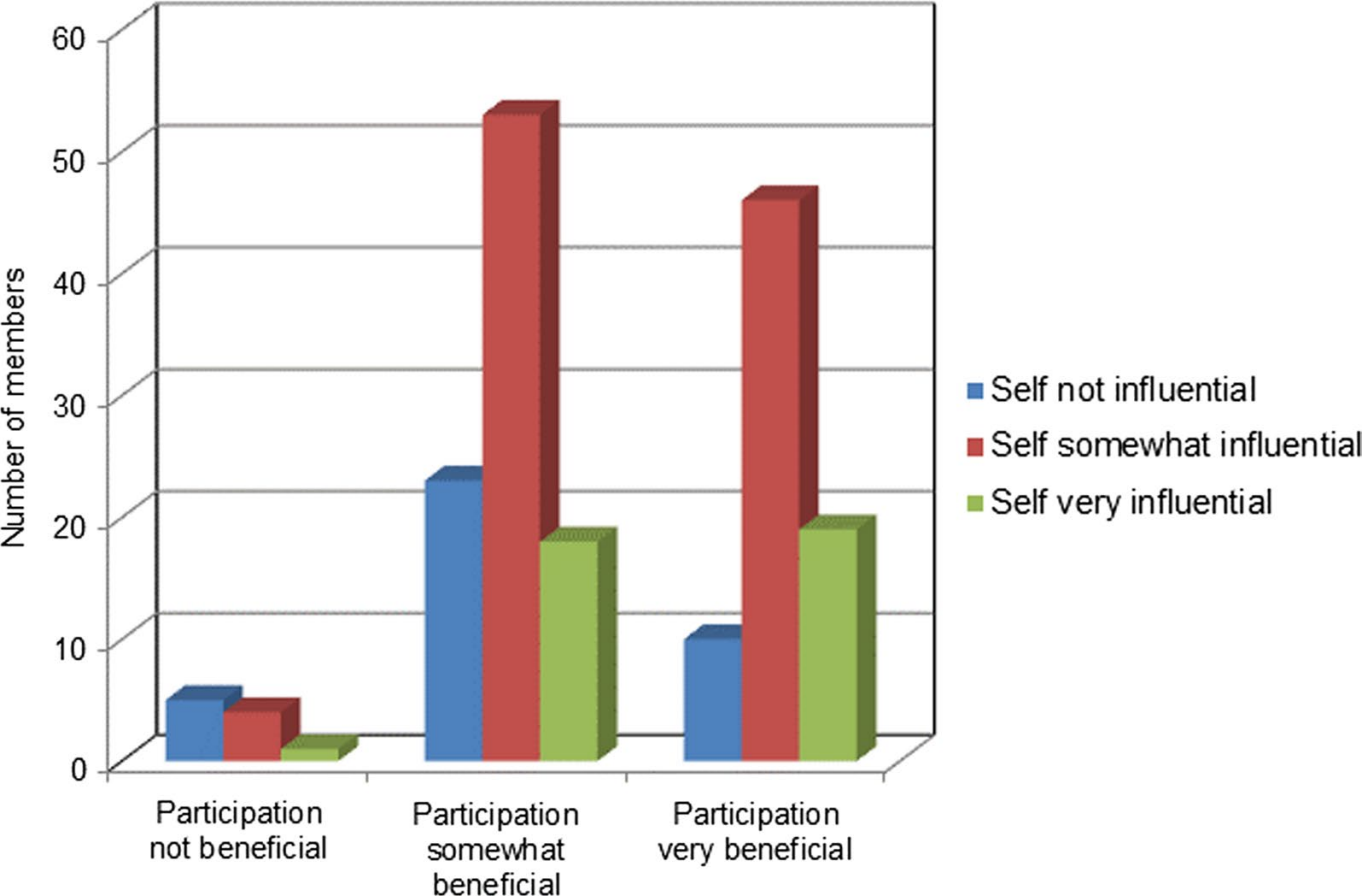
Benefit of participation for self-reported influential members

Respondents were also asked, overall, whether they considered their participation in the HF Network to be beneficial, and how influential they were in making changes in the quality of care of HF patients at their site. Figure 6 shows that almost all the respondents (97%) perceived their participation in the HF Network as beneficial. Among respondents, 19.6% perceived themselves as highly influential, 55.6% as somewhat influential, and 24.8% as not influential in making changes in the quality of care of HF patients at their own site. Similarly, most respondents perceived their participation in the HF Network as somewhat beneficial (51.6%) or very beneficial (42.9%). Interestingly, those respondents who considered themselves influential in making changes in the quality of care of HF patients at their site also found their participation more beneficial than those who perceived themselves as not influential.

#### Phone interview findings

A total of 18 semi-structured interviews were conducted with physicians (*n* = 10), nurses (*n* = 7) and VACO/VISN leaders (*n* = 1). Qualitative analysis from the semi-structured interviews shows that these participants perceived the goals of the HF Network as sharing information (*n* = 11), improving care for HF patients (*n* = 10) and providing information based on EBPs (*n* = 5). They participated in the HF Network to stay informed (*n* = 10), maintain/enhance their knowledge (*n* = 8) and collaborate with other members (*n* = 5). HF Network activities helpful to these participants were discussions about setting up HF programmes (*n* = 11), discussions focusing on QI projects (*n* = 4) and collaborations with the HF Network (n = 6). They considered the HF Network as “…a good tool for networking” and “meeting potential collaborators to share information about current research”.

Most participants said they had no concerns related to the HF Network (*n* = 11) and they had referred other members to join the HF Network (*n* = 11). Many of them perceived themselves as having influence in making changes at their own site (*n* = 13).

At the site-level, the barriers to success of the HF Network were reported as limited time (*n* = 8) and lack of resources (*n* = 4), and facilitators for the success of the HF Network were commitment and support (*n* = 4).

### Site level

#### Participation levels

Among those who responded to the survey (*n* = 219), Table 5 shows the comparison of the site characteristics based on the categorization of member-level participation in the activities of the HF Network from year 1 through year 4. Analysis of variance (ANOVA) showed that these members belonged to 124 sites and they participated at three levels: none/low (*n* = 47; 0–5 activities), moderate (*n* = 36; 6–10 activities) and high (*n* = 41; 11 ≥ activities). These differences were significant at *P* > 0.001 level.

**Table 5:** Characteristics of Sites Based on Site Participation Level

Site characteristics	Site-level participation by members			
	None/low (*N* = 39) *N* (%)	Moderate (*N* = 61) *N* (%)	High (*N* = 119) *N* (%)	*P* value
Tertiary care site	13 (5.9)	38 (17.4)	78 (35.6)	0.002**
Bed size				
1–99 beds	10 (4.8)	5 (2.4)	13 (6.3)	0.01**
100–199 beds	7 (3.4)	18 (8.7)	17 (8.2)	
200 or more beds	19 (9.2)	36 (17.4)	82 (39.6)	
Member COTH (Council of Teaching Hospitals)	14 (6.7)	32 (15.2)	62 (29.5)	0.250
Has ACGME (Accredited Graduate Medical Education) programme	28 (13.3)	53 (25.2)	110 (52.4)	0.005**
Has a cardiac cath lab	36 (16.4)	58 (26.5)	119 (54.3)	0.01**
Has on-site cardiologist services	35 (16.0)	61 (27.9)	117 (53.4)	0.005**
Has a HF clinic	20 (9.1)	50 (22.8)	87 (39.7)	0.004**
Use of pharmacist	27 (12.3)	45 (20.5)	84 (38.4)	0.860
Standardized home monitoring	29 (13.2)	55 (25.1)	98 (44.7)	0.110

*P value significant at 0.05 level; **P value significant at 0.01 level

#### Quality indicators

Categorizing member-level participation in the activities of the HF Network from year 1 through year 4, using one-way ANOVA, Table 6 shows a comparison of means for the three levels of site-level participation by the members (*n* = 219). As is evident, these groups of sites differed significantly from each other (*p* value ≤ 0.001). Among these three groups of sites, the level of member participation did not significantly impact 30-day mortality after admission (*p* = 0.225). But both death at 1 year after admission (*p* = 0.245) and all-cause readmission after 30 days (*p* = 0.005) were found to be significantly different, with groups of sites where members were highly active participants having higher readmission rates.

**Table 6:** Average site quality indicators based on site-level participation by members

Site characteristics during years 2–7	Site-level participation by members			
	None/low Mean (SD) *N* = 47 (37.9%)	Moderate Mean (SD) *N* = 36 (29.0%)	High Mean (SD) *N* = 41 (33.1%)	*P* value
ACE inhibitor	0.963 (0.057)	0.963 (0.047)	0.979 (0.021)	0.198
Beta-blocker use*	0.940 (0.067)	0.946 (0.047)	0.952 (0.044)	0.565
Use of aldosterone antagonist*	0.230 (0.146)	0.254 (0.122)	0.275 (0.124)	0.301
Death 30 days after admission*	0.068 (0.028)	0.073 (0.031)	0.062 (0.023)	0.225
Death at 1 year after admission*	0.297 (0.068)	0.285 (0.051)	0.278 (0.040)	0.245
30-Day all-cause readmission following discharge with principal diagnosis of HF	0.165 (0.041)	0.177 (0.038)	0.191 (0.024)	0.005

*Trends analysis

We also compared levels of member participation in these 3 groups of sites for the following processes of care. Higher participating sites were significantly better in the use beta blockers (*p* = 0.565) and ACE inhibitors (*p* = 0.198) but not regarding the use of aldosterone antagonist (*p* = 0.301). While trends analysis for all these three processes of care were not significant for beta blocker (*p* = 0.881) and ACE inhibitor (*p* = 0.501) and only borderline for aldosterone antagonist (*p* = 0.087), these trends were indicative of care in the right direction.

### Impact of the formative evaluation on CoP

Based on the results of the formative evaluation, several changes were made to the HF Network. First, given the rich mix of clinicians and non-clinicians attending each meeting, we now include two presentations for each call, with one focusing on a research project and the other on a QI initiative. Second, given the importance that members placed on the role of champions, we sought local champions to serve as “internal” facilitators at each site. We were able to identify such champions at 65% of the sites. Third, given the many responses indicating that the HF Network can be helpful “…to keep as updated as possible with the standards for treating the HF patients”, we developed a web-based Heart Failure Toolkit for the members of the HF Network as well as other VA providers [20]. This toolkit focuses on several key areas in the management of heart failure, with downloadable documents.

## Discussion

The purpose of this study was to describe a formative evaluation of the CoP (HF Network). The main observation is that members, particularly those that consider themselves influential in improving quality of care, have noted multiple benefits of participating in the HF Network.

The major strength of the HF Network lies in its multidisciplinary and multilevel membership. Over two thirds of these participants have been actively participating in the HF Network activities. Their participation helped them validate their own current practice in taking care of patients, encouraged evidence-based changes in practice and helped solve implementation-related problems. Given the extremely high VA’s web traffic data measuring use of the HF Network website, anecdotal evidence and reported barriers to active participation such as limited time and lack of resources, we are confident that most of the remaining members have been “passively” participating by viewing/downloading resources and networking/collaborating with other members.

There is strong evidence of the sustainability of this network over the 9 years, as membership saw a steady sixfold increase over this period. Then, as is typical in any organization, there was also attrition as some members left the HF Network while other members left the VA itself.

The wide variety of resources in terms of activities of the HF Network was perceived as helpful by members. Our findings also show sharing of HF as a concern involving identity-building and networking (social interactions) with deepening of knowledge and expertise through interactions and helping one another by both active and passive participation on an ongoing basis. Strong influences of their participation was evident in terms of self-reported validation of their own current practice in taking care of HF patients, evidence-based changes in their practice and help in understanding facilitators and barriers in setting up or running HF clinics/programmes. This observation was shared in a review of the literature, which found that CoPs were promoted in the healthcare sector as a means of generating and sharing knowledge and improving organizational performance [13].

The present study revealed an interesting association between member-reported self-influence in making changes in the quality of care of HF patients at own site and benefit of own participation in the HF Network. Those members who considered themselves influential in making changes in the quality of care of HF patients at their site also found their participation to be very beneficial. This finding has important implications, as we expect that influential members who find their participation beneficial would be among those who reported that their participation in the HF Network helped solve an implementation-related problem at their own site, helped influence leadership/administration at their own site to improve HF care, and helped influence other members/staff to improve HF care. These strong findings substantiate the two complementary theoretical approaches being used to guide the implementation of interventions through the HF Network. Based on Rogers’ diffusion of innovation theory [25], we have used local opinion leaders in shepherding the implementation efforts. Also, based on the Promoting Action on Research Implementation in Health Services (PARIHS) framework’s [26] “facilitation” element, we have used a “blended” facilitation approach to implement HF-related EBPs at the local, regional and/or national levels. It should be noted that members with senior roles may be more effective in implementation and more socially inclined than other members, and thus, networks with greater or fewer members with senior roles may have different outcomes.

On a similar note, the qualitative findings also support both these theoretical frameworks. The role of champions as stated by Rogers [26] found strong empirical support here. A nurse described a facilitator for the success of the HF Network: “I think having a champion, I think multi-departmental buy-in, and I think you also need top administration to support it. Those three things”. Another nurse said, “I have no problem with taking that hour because the chief of cardiology is one of the major supporters of the CHF thing”.

The main strength of the CoP theory is that it is able to provide a basis for the development and delivery of theory-informed implementation interventions as well as their evaluation, which is especially important in the current situation when theory is not sufficiently utilized in the field of implementation research [27]. Utilizing Rogers’ diffusion of innovation theory [25] and the PARIHS framework [26], the HF Network has been used both as a mechanism to implement interventions (research and QI projects) and as a vehicle to obtain funding for implementation-focused research and QI projects.

An important aspect is assessing the value of the HF Network as a CoP to the participating and nonparticipating members and key stakeholders including VACO leadership. The success of this organically grown HF Network with careful management and rigorous evaluation encourages the growth of similar CoPs through the development of social networks within the VA healthcare system for other conditions. In this VA context, CoPs focusing on issues relating to returning Veterans and sexual trauma would need to expand the key stakeholders by including the patients and caregivers. CoPs are interesting structures to facilitate intra- and interdisciplinary collaborations necessary to accelerate the implementation of the chronic disease model and best practice recommendations [28]. Our effort can facilitate the establishment and effectiveness of similar networks within VA and contributes new insights and evidence regarding the operation and impacts of CoPs (and similar social network strategies) in improving healthcare quality and outcomes and facilitating implementation of EBPs and innovations in healthcare delivery.

We developed the CoP for HF care as a social network. This was based on the rationale that social networks are an important source of tacit knowledge, and thus there is growing interest in the use of networks to facilitate knowledge exchange in healthcare settings. Their structures provide opportunities and incentives to their members along with a high degree of connectedness which enhances imitation of behaviours and related social processes, resulting in more homogeneous practice patterns [29]. As noted by Mittman and colleagues, healthcare professionals work within peer groups who often share common values, assumptions and beliefs, and professional practices can be strongly influenced by these factors [30]. There is strong evidence that physicians obtain information and related guidance (e.g. professional norms, values, attitudes) from other physicians whom they consider to be peers and to possess expertise in the knowledge area [31].

A study by Palinkas and colleagues [32] found that the structure and operation of social networks were central to implementation of EBPs. Further, social networks influence the implementation process through two mechanisms: development and operation of successful collaborations, and acquisition of information and support related to EBPs. Within the United States VA, Parchman and colleagues [33] examined the properties of a network created by "co-care” of patients within one region. They found that the network was complex, consisting of highly connected provider nodes that serve as "hubs" within the network, and demonstrating some "scale-free” properties.

In Canada, Conklin and Stolee [34] focused on understanding the processes mobilized through various CoPs that are working to improve health in the Seniors Health Research Transfer Network (SHRTN) in Ontario. They found that the CoP functioned as an incubator that brought together best practices, research, experiences, a reflective learning cycle and passionate champions. Also in Canada, Norman and Huerta [35] examined building foundations for a CoP using evaluation and social network methodologies. A well-designed evaluation protocol was reported by Conklin et al. [36] using the PARIHS framework [26], with a shift in focus towards frontline practices where it was hoped to be implemented. In a previous study, Conklin and Stolee’s [34] evaluation model efforts showed that SHRTN and the CoP provide a supportive context but that continued active facilitation of knowledge exchange is necessary at the point of care. In 2010, Poissant et al. [28] reported that emergent CoPs within Canada’s Montreal Stroke Network were successful in developing and implementing critical inputs such as referral tools that accelerated patient transition between acute care and rehab. A later study [37] demonstrated that knowledge brokers who support a CoP take on a complex and demanding role in supporting the development of the CoP, assisting with specific initiatives and promoting the growth of the network. Moreover, their role is contextual. Supporting the development of a new CoP differs from supporting the efforts of a well-established CoP.

One remarkable finding was the higher rate of hospitalization in those sites that were more engaged with the HF Network. There are several potential reasons for this counterintuitive finding. Prior studies have shown that improved access to outpatient care and availability of better specialty care were both associated with higher hospitalizations rates for conditions that included HF [38, 39]. This occurs because HF admissions are often borderline elective. These studies show that providers often appropriately recognize a need for admission that the patient does not. Thus, having more expert providers with greater patient access can paradoxically increase admissions for HF.

There are several policy implications of this work. Health research is conducted with the expectation that it advances knowledge and eventually translates into improved health systems and population health [40]. Haynes and colleagues postulated that health policy-making could benefit from more effective use of research. Their exploratory review tentatively posited causal mechanisms that might explain how intervention strategies work in different contexts to build capacity for using research in policy-making [41]. Research by Auer et al. highlights the potential for CoPs to influence practice and broad-scale change more directly than previously understood or reported in the literature [5]. Their systematic reviews and clinical practice studies [12] have highlighted significant opportunities for CoPs to substantially influence healthcare. CoPs have come to be recognized as vehicles for important healthcare system advancements such as increasing integration between primary and tertiary care to reduce unnecessary referrals [42], promoting the adoption of change to improve the care of seniors [6] and improving the uptake of care practices and new practitioner mentoring in public health [43]. The study organization recognizes CoPs as learning and capability assets in its employee development resources, a link also seen in best-practice organizations studied by the American Productivity & Quality Center [44].

Despite a rapidly growing body of literature about the use of research in policy-making, we have a limited understanding of how best to help policy-makers use research in their day-to-day work, partly because most of the literature is either descriptive or theoretical [41]. A recent review of studies of multi-programme interventions by Hanney et al. found many reported impacts on policy, practice and health services research, demonstrating a wide variety of interventions [45]. There are many challenges facing health research, including securing sufficient funds, building capacity, producing research findings, using both local and global evidence and avoiding waste [46]. It has been noted that there is little evidence to guide efforts to increase the use of evidence in policy [46], despite this use being a common government priority. Many promising technological innovations in health and social care are characterized by non-adoption or abandonment by individuals or by failed attempts to scale up locally, spread distantly or sustain the innovation over the long term at the organization or system level [47]. Our study shows how these barriers can be addressed through the creation of a CoP. The emergence of CoPs in healthcare help realize the full potential of EBPs to optimize health and healthcare by bridging the gap between QI initiatives, research, practice and policy. The involvement of multilevel and multidisciplinary members of the sustained CoPs provides extended opportunities to share explicit and tacit knowledge across the organization, create a culture of learning and knowledge translation, and prioritize EBPs to inform decisions. This study demonstrates that with limited facilitation, a robust CoP can be created across more than 100 sites within a national healthcare system. Such healthcare systems can create CoPs to cover high-priority clinical areas and use them in concert with other strategies to improve quality such as electronic reminders, educational campaigns, and audit and feedback interventions.

This study has several potential limitations. A major limitation is that we were unable to link the survey responses to the respondent’s role, type of member or site name. As was noted above, the HF Network membership grew steadily over the 9 years, with “active” (70.6%) versus “passive” (29.4%) members having opportunities to participate in the four specific activities for which participation was being tracked. The survey was emailed to 878 individuals who had been members of the HF Network for at least 6 months, irrespective of their active versus passive participation status. This was a voluntary cross-sectional survey with the expectation that the “passive” members in particular may not have been likely to complete the survey. Since members were asked to participate in any one or more activities that met their interest and needs over varying periods of time, with time understandably being a potential constraint, we decided to keep the survey anonymous (delinked) so that respondents completing the survey would provide their best responses. Therefore, the survey data had to be analysed at the general level instead of allowing a comparison of responses based on the respondent’s role and type of site. If linked survey data were available for all participating members, then it would have been interesting to see how perceptions vary based on their roles and both academic and site characteristics. Further, though the survey had a low response rate of 24.9% (*n* = 219), the roles of the respondents were consistently representative of the roles of the total HF Network members. As stated earlier, the highest representation was observed for the practising clinicians, followed by administration, and then researchers and others. At the time of the formation and evaluation of the HF Network, formal cardiac rehabilitation was rarely used for HF patients. Thus, we did not have a significant number of cardiac physiologists or physical therapists in the HF Network.

## Conclusions

We established the HF Network as a CoP for VA members to facilitate networking, information dissemination and exchange, and collaboration among VA HF members to improve HF care for Veterans. Several hundred multidisciplinary members and administrators from throughout the VA continue to join and participate as members of the HF Network. Strong evidence in varied forms supports the contention that these members perceive the HF Network as useful in terms of its varied activities and resources relevant for patient care. Members, particularly those who consider themselves influential in improving quality of care, have noted multiple benefits of membership. Such CoPs have strong potential for increasing medical knowledge among providers, spreading best practices across health systems and improving outcomes for patients.

## Data Availability

This study has two types of datasets, and neither of them is publicly available. The first dataset generated for the study contains sensitive personal health information (first and last names, email addresses, location, and roles) and participation of the VA employees in the HF Network. The second dataset was generated based on VA’s highly sensitive and protected administrative datasets which contains site characteristics and site quality indicators.

## Abbreviations

CoPs: Communities of Practice
PARiHS: Promoting Action on Research Implementation in Health Services
EBP: Evidence-based practice
HF: Heart failure
HF Network: Heart Failure Provider Network
QI: Quality improvement
VA: United States Department of Veterans Affairs
VACO: Veterans Affairs Central Office
VISN: Veterans Integrated Service Network
CIDER: Center for Information Dissemination and Education Resources
